# Pathophysiology and treatment of atherosclerosis: Current view and future perspective on lipoprotein modification treatment

**DOI:** 10.1007/s12471-017-0959-2

**Published:** 2017-02-13

**Authors:** S. C. Bergheanu, M. C. Bodde, J. W. Jukema

**Affiliations:** 1 Centre for Human Drug Research, Leiden, The Netherlands; 2 InterEuropa Clinical Research, Rotterdam, The Netherlands; 3 Department of Cardiology C5-P, Leiden University Medical Center, Leiden, The Netherlands

**Keywords:** Atherosclerosis, Hypercholesterolaemia, Low-density lipoprotein, Cardiovascular disease, Statins, Proprotein convertase subtilisin/kexin type-9

## Abstract

Recent years have brought a significant amount of new results in the field of atherosclerosis. A better understanding of the role of different lipoprotein particles in the formation of atherosclerotic plaques is now possible. Recent cardiovascular clinical trials have also shed more light upon the efficacy and safety of novel compounds targeting the main pathways of atherosclerosis and its cardiovascular complications.

In this review, we first provide a background consisting of the current understanding of the pathophysiology and treatment of atherosclerotic disease, followed by our future perspectives on several novel classes of drugs that target atherosclerosis. The focus of this update is on the pathophysiology and medical interventions of low-density lipoprotein cholesterol (LDL-C), high-density lipoprotein cholesterol (HDL-C), triglycerides (TG) and lipoprotein(a) (Lp(a)).

Atherosclerosis is a chronic condition in which arteries harden through build-up of plaques. Main classical risk factors for atherosclerosis include dyslipoproteinaemia, diabetes, cigarette smoking, hypertension and genetic abnormalities. In this review, we present an update on the pathophysiology of atherosclerosis and related current and possible future medical interventions with a focus on low-density lipoprotein cholesterol (LDL-C), high-density lipoprotein cholesterol (HDL-C), triglycerides (TG) and lipoprotein(a) (Lp(a)).

## Pathophysiology of atherosclerosis

Hypercholesterolaemia is considered one of the main triggers of atherosclerosis. The increase in plasma cholesterol levels results in changes of the arterial endothelial permeability that allow the migration of lipids, especially LDL-C particles, into the arterial wall. Circulating monocytes adhere to the endothelial cells that express adhesion molecules, such as vascular adhesion molecule-1 (VCAM-1) and selectins, and, consequently, migrate via diapedesis in the subendothelial space [1]. Once in the subendothelial space, the monocytes acquire macrophage characteristics and convert into foamy macrophages. LDL particles in the subendothelial space are oxidised and become strong chemoattractants. These processes only enhance the accumulation of massive intracellular cholesterol through the expression of scavenger receptors (A, B1, CD36, CD68, for phosphatidylserine and oxidised LDL) by macrophages, which bind native and modified lipoproteins and anionic phospholipids. The end result is a cascade of vascular modifications [1] described in Table 1. Clinical sequelae of atherosclerosis are vessel narrowing with symptoms (angina pectoris) and acute coronary syndromes due to plaque instability.

**Table 1:** Vascular modifications in atherosclerotic disease

Vascular modification	Characteristics
Intimal thickening	Layers of SMCs and extracellular matrix More frequent in coronary artery, carotid artery, abdominal aorta, descending aorta, and iliac artery
Fatty streak	Abundant macrophage foam cells mixed with SMCs and proteoglycan-rich intima
Pathologic intimal thickening	Layers of SMCs in proteoglycan-collagen matrix aggregated near the lumen Underlying lipid pool: acellular area rich in hyaluronan and proteoglycans with lipid infiltrates
Fibroatheromas	Acellular necrotic core (cellular debris) Necrotic core is covered by a thick fibrous cap: SMCs in proteoglycan-collagen matrix
Vulnerable plaque	‘Thin-cap fibroatheroma’ Type I collagen, very few/absent SMCs Fibrous cap thickness is ≤65 µm
Ruptured plaque	Ruptured fibrous cap Presence of luminal thrombus Larger necrotic core and increased macrophage infiltration of the thin fibrous cap

*SMCs* smooth muscle cells

The majority of coronary thrombi are caused by plaque rupture (55–65%), followed by erosions (30–35%), and least frequently from calcified nodules (2–7%) [1]. Rupture-prone plaques typically contain a large, soft, lipid-rich necrotic core with a thin (≤65 µm) and inflamed fibrous cap. Other common features include expansive remodelling, large plaque size (>30% of plaque area), plaque haemorrhage, neovascularisation, adventitial inflammation, and ‘spotty’ calcifications. Vulnerable plaques contain monocytes, macrophages, and T‑cells. T‑cells promote the vulnerability of plaques through their effects on macrophages [2].

LDL-C, TG and HDL-C emerged as strong independent predictors of atherosclerotic disease after the analysis of the data from the Framingham study. While the role of other parameters is being investigated, TC, LDL-C and HDL-C remain to date the cornerstone in risk estimation for future atherosclerotic events. Low HDL-C has been shown to be a strong independent predictor of premature atherosclerosis [3] and is included in most of the risk estimation scores. Very high levels of HDL-C, however, have consistently not been found to be associated with atheroprotection. The mechanism by which HDL-C protects against atherosclerosis is still under debate and accumulating evidence strongly suggests that the proportion of dysfunctional HDL versus functional HDL rather than the levels may be of importance.

Hypertriglyceridaemia (HTG) has been shown to be an independent risk factor for cardiovascular disease (CVD). Moreover, high TG levels are often associated with low HDL-C and high levels of small dense LDL particles. The burden of HTG is high, with about one-third of adult individuals having TG levels >1.7 mmol/l (150 mg/dL) [3].

Lp(a) is a specialised form of LDL and consists of an LDL-like particle and the specific apolipoprotein (apo) A. Elevated Lp(a) is an additional independent risk marker and genetic data made it likely to be causal in the pathophysiology of atherosclerotic vascular disease and aortic stenosis [4].

## Lipoprotein modification treatment

### Current view

Medication to adequately control lipoprotein levels needs to be initiated when risk reduction through lifestyle modifications such as dietary changes, stimulation of physical activity and smoking cessation is not sufficient. In secondary prevention, medical therapy is almost invariably needed in addition to lifestyle optimisation.

#### LDL-C-lowering therapy

##### HMG-CoA reductase inhibitors (statins)

3-hydroxy-3-methyl–glutaryl-coenzyme A (HMG-CoA) reductase inhibitors (usually addressed as ‘statins’) induce an increased expression of LDL receptors (LDL-R) on the surface of the hepatocytes, which determines an increase in the uptake of LDL-C from the blood and a decreased plasma concentration of LDL-C and other apo B‑containing lipoproteins, including TG-rich particles [3].

Since the 1990s, statin therapy has shown its effect on cardiovascular outcome in several major landmark trials, summarised in Table 2.

**Table 2:** Summary of major clinical trials and programs involving low-density lipoprotein cholesterol lowering treatments

Drug/Target	Clinical trial	Study size	Duration	CV endpoints	Results
Statins	4 S [44]	4444 patients with CHD	5.4 y	Coronary death	111 in the simvastatin group; 189 in the placebo group; (RR = 0.58, 95% CI: 0.46–0.73)
	WOSCOP [45]	6595 men with hypercholesterolemia	4.9 y	Combined nonfatal MI/coronary death	174 in the pravastatin group; 248 in the placebo group; (RRR = 31%, 95% CI: 17–43%)
	CARE [46]	4159 subjects with high CV risk and normal LDL-C levels	4.9 y	Combined coronary event/nonfatal MI	10.2% in the pravastatin group; 13.2% in the placebo group; (RRR = 24%, 95% CI: 9–36%)
	ASTEROID [47]	349 patients on statin therapy with serial IVUS examinations	2.0 y	IVUS change in PAV	−0.79% (−1.21 to −0.53%) in the rosuvastatin group
	SATURN trial [48]	1039 patients with CAD on intensive statin treatment	2.0 y	IVUS change in PAV	−0.99% (−1.19 to −0.63%) in the atorvastatin group; −1.22% (−1.52 to −0.90%) in the pravastatin group
	REGRESS [9]	885 symptomatic male patients on pravastatin or placebo	2.0 y	Change in lumen diameter	0.10 mm decrease in the placebo group; 0.06 mm decrease in the pravastatin group (*p* = 0.019)
	PROVE-IT TIMI 22 [10]	4162 ACS patients on either intensive or standard statin therapy	2.0 y	Combined death, MI, UAP, revascularization, stroke	22.4% in intensive therapy group; 26.3% in standard statin therapy group; (HR 0.84, 95% CI: 0.74–0.95)
Ezetimibe	PRECISE-IVUS [14]	246 patients undergoing PCI on statin alone or statin + ezetimibe	9.9 m	IVUS change in PAV	−1.4% (−3.4 to −0.1%) in the dual lipid lowering group; −0.3% (−1.9 to 0.9%) in the statin monotherapy group
	IMPROVE-IT [15]	18,114 ACS patients on statin + placebo or on statin + ezetimibe	6.0 y	Combined death, MI, UAP, revascularization, stroke	32.7% in simvastatin + ezetimibe group; 34.7% in the simvastatin + placebo group; (HR 0.94, 95% CI: 0.89–0.99)
Bile acid sequestrants	LRC-CPP [49]	3806 men with hypercholesterolemia on cholestyramine resin or placebo	7.4 y	Combined CAD death/nonfatal acute MI	8.1% in cholestyramine group; 9.8% in the placebo group; (RR 0.81, 90% CI: 0.68–0.84)
PCSK-9 inhibitors	OSLER [16]	4465 patients on evolocumab + standard therapy or standard therapy alone	11.1 m	%change LDL-C, cardiovascular events	−61% (−59 to −63%) LDL-C change in the evolocumab group, 0.95% event-rate in the evolocumab group; 2.18% in the standard therapy group; (HR 0.47, 95% CI 0.28–0.78)
	ODYSSEY LONG TERM [17]	2341 high risk patients receiving in a 2:1 ratio alirocumab or placebo	78 w	%change in LDL-C, combined death, MI, UAP, revascularization, stroke	−61% LDL-C change in the alirocumab group; 0.8% in the placebo group; (*p* < 0.001). 1.7% event-rate in the alirocumab group; 3.3% in the placebo group; (HR 0.52, 95% CI: 0.31–0.90)
	GLAGOV [18]	968 presenting for CAG randomized with either evolocumab or placebo	76 w	IVUS change in PAV	−1.0% (−1.8 to −0.64%) in the evolocumab group

*CHD* coronary heart disease, *CAD* coronary artery disease *MI* myocardial infarction, *CV* cardiovascular risk, *LDL-C* low-density lipoprotein cholesterol, *PAV* percentage atheroma volume, *ACS* acute coronary syndrome, *PCI* percutaneous coronary intervention, *UAP* unstable angina pectoris, *CAG* coronary angiography, *IVUS* intravascular ultrasonography, *y* year, * m* months, *RR* relative risk, *HR* hazard ratio, *CI* confidence interval, *4S* Scandinavian Simvastatin Survival Study, *WOSCOP* West of Scotland Coronary Prevention, *CARE* Cholesterol and Recurrent Events, *ASTEROID* A Study to Evaluate the Effect of Rosuvastatin on Intravascular Ultrasound – Derived Coronary Atheroma Burden, *SATURN* The Study of Coronary Atheroma by Intravascular Ultrasound: Effect of Rosuvastatin versus Atorvastatin, *REGRESS* The Regression Growth Evaluation Statin Study, *REVERSAL* Reversal of Atherosclerosis with Aggressive Lipid Lowering, *PROVE-IT TIMI 22* pravastatin or atorvastatin evaluation and infection trial-thrombolysis in myocardial infarction, *PRECISE-IVUS* Plaque Regression With Cholesterol Absorption Inhibitor or Synthesis Inhibitor Evaluated by Intravascular Ultrasound, *IMPROVE-IT* IMProved Reduction of Outcomes: Vytorin Efficacy International Trial, *LRC-CPP* Lipid Research Clinics Coronary Primary Prevention, *OSLER* open-label study of long-term evaluating against LDL-C, *ODYSSEY LONG TERM* Long-term Safety and Tolerability of Alirocumab in High Cardiovascular Risk Patients with Hypercholesterolemia Not Adequately Controlled with Their Lipid Modifying Therapy, *GLAGOV* global assessment of plaque regression with a PCSK-9 antibody as measured by intravascular ultrasound

**Fig. 1:**
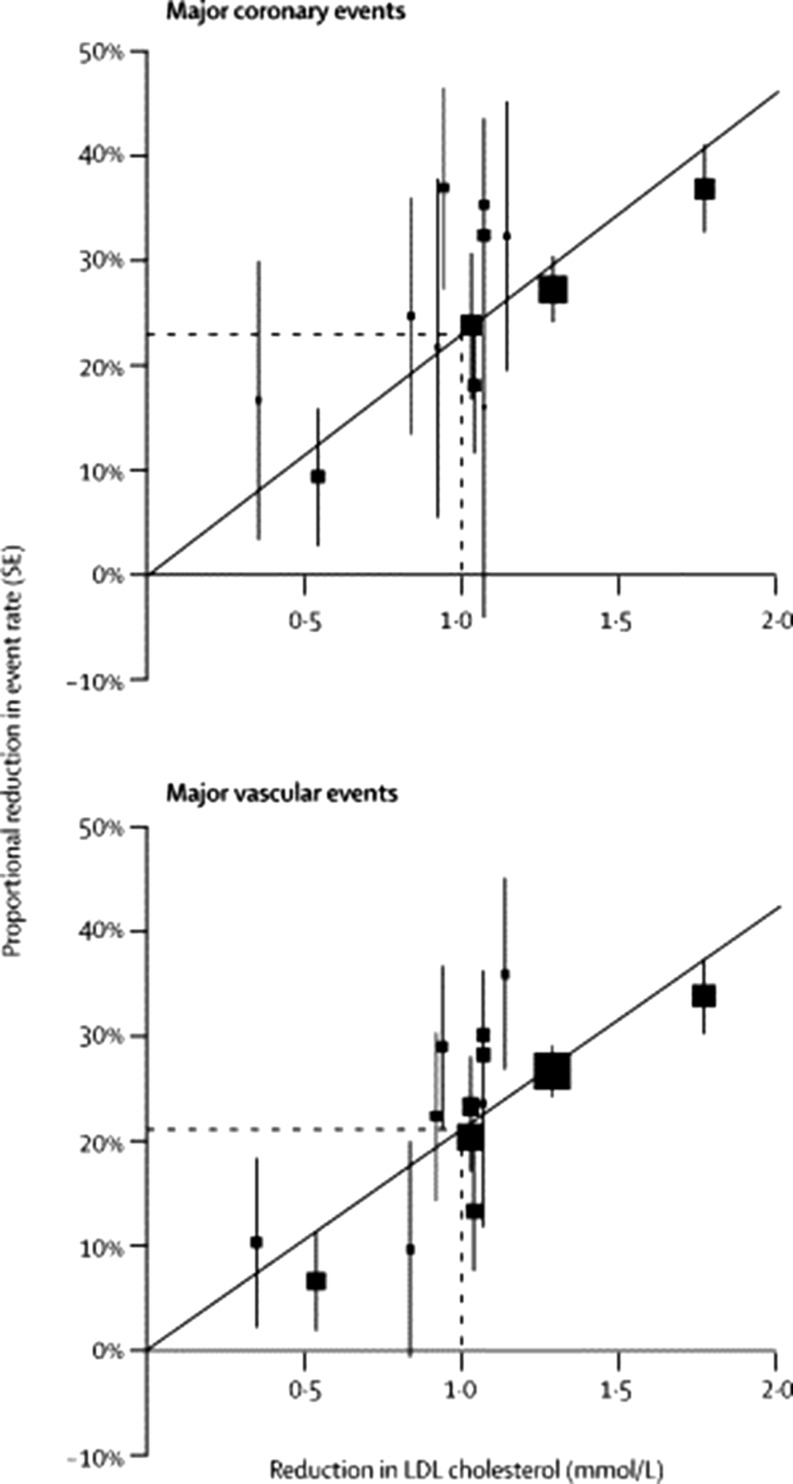
Relation between proportional reduction in incidence of major coronary events and major vascular events and mean absolute LDL cholesterol reduction at 1 year. Square represents a single trial plotted against mean absolute LDL cholesterol reduction at 1 year, with vertical lines above and below corresponding to one SE of unweighted event rate reduction. Trials are plotted in order of magnitude of difference in LDL cholesterol difference at 1 year. For each outcome, regression line (which is forced to pass through the origin) represents weighted event rate reduction per mmol/l LDL cholesterol reduction. (Figure published with permission of the Lancet (owned by Elsevier))

Independent of baseline LDL-C level and baseline cardiovascular (CV) risk, meta-analyses concerning up to 27 statin CV outcome trials, showed a 22% risk reduction in CV events per 1 mmol/l reduction in LDL-C ([5–7]; Fig. 1).

It is currently known that both the baseline burden of atherosclerotic plaque and the degree of progression on serial evaluation significantly associate with risk of CV events [8, 9]. The difference in change in percent atheroma volume (PAV) between patients with and without an event can be as low as approximately 0.55% [10].

Not reaching the cholesterol treatment goals and non-compliance are two important causes for statin therapy failure. Although the LDL-C levels obtained in clinical trials are often low, the clinical reality seems different. Vonbank et al. [11] showed that in 2 cohorts of high-risk CV patients, one from 1999–2000 and the other one from 2005–2007, only 1.3% and 48.5% of patients, respectively, had the LDL-C < 1.8 mmol/l at 2‑year follow-up. The fear of possible side effects of statin therapy is an important reason for non-compliance and remains an underestimated problem in clinical practice. One study in high-dose statin patients reported that muscular pain prevented even moderate exertion during everyday activities in 38% of patients, while 4% of patients were confined to bed or unable to work [12]. Jukema et al. reviewed available data and concluded that statin use is associated with a small increase in type 2 diabetes mellitus incidence, but no convincing evidence was found for other major adverse effects such as cognitive decline or cancer [13].

Statins are therefore, in general, very efficient drugs that in an overwhelming amount of well conducted clinical trials showed consistent clinical event reductions with a very good safety profile. Nevertheless, side effects of importance may occur making the compound, as in any drug class, sometimes unsuitable for some individual patients.

##### Cholesterol absorption inhibitors

By inhibiting cholesterol absorption, ezetimibe reduces LDL-C. In clinical studies, ezetimibe as monotherapy reduced LDL-C by 15–22% and when combined with a statin it induced an incremental reduction in LDL-C levels of 15–20% [3]. No frequent major adverse effects have been reported [3]. Results from studies like PRECISE-IVUS [14] and IMPROVE-IT [15] support the use of ezetimibe as second-line therapy in association with statins when the therapeutic goal is not achieved at the maximum tolerated statin dose, in statin-intolerant patients, or in patients with contraindication to statins [3].

##### Bile acid sequestrants

At the highest dose, cholestyramine, colestipol or the recently developed colesevelam can produce a reduction in LDL-C of 18–25% [3]. The use of cholestyramine and colestipol is limited by gastrointestinal adverse effects and major drug interactions with other frequently prescribed drugs. Colesevelam appears to be better tolerated and to have less interaction with other drugs and can be combined with statins. Relatively little hard evidence is available from large clinical trials for this class of drugs.

##### Proprotein convertase subtilisin/kexin type-9 inhibitors

Inhibitors of proprotein convertase subtilisin/kexin type-9 (PCSK-9) offer the prospect of achieving even lower LDL-C levels than statins in combination with ezetimibe. PCSK-9 binds to LDL-R at the liver and stimulates the absorption and degradation of these receptors. Through inhibition of PCSK-9, the degradation of LDL-R is prevented thereby improving the absorption by the liver of LDL-C particles, which consequently leads to lower LDL-C plasma concentrations.

In 2015, reports were published from two phase 3 trials that measured the efficacy and safety of evolocumab and alirocumab, two monoclonal antibodies that inhibit PCSK-9 [16, 17]. In these trials, the PCSK-9 therapy significantly lowered LDL-C by ≈ 50% and in a preliminary (not powered) analysis reduced the incidence of CV events (Table 3). Other promising results were published from the GLAGOV [18] trial and demonstrated a significant percentage atheroma volume decrease with evolocumab (Tab﻿le 3). Both evolocumab and alirocumab have been recently approved by the European Medicine Agency and the US Food and Drug Administration for the treatment of elevated plasma LDL-C. The PCSK-9 therapy is suitable in a wide range of patients provided that they express LDL-R, including those with heterozygous and homozygous familial hypercholesterolaemia with residual LDL-R expression [3]. Relatively high costs of the compounds and yet the lack of hard outcomes in large randomised controlled trials (RCTs) still limit their use in clinical practice.

**Table 3:** Trials concerning PCSK-9 inhibition

Clinical trial	Mechanism of action	Molecules	Population	Phase	Endpoint	Expected/known results
ODYSSEY OUTCOME [19]	PCSK-9 antibodies	Alirocumab	18,000 post ACS patients	3	Combined CAD death/nonfatal acute MI	2017/2018
FOURIER [20]	PCSK-9 antibodies	Evolocumab	27,564 high risk patients with LDL-C > 1.8 mmol/L	3	Combined CAD, death/nonfatal acute MI	Early 2017
SPIRE 1 + 2 [21]	PCSK-9 antibodies	Bococizumab	28,000 patients on high residual risk	3	Combined death, MI, UAP, revascularization, stroke	Terminated due to the emerging clinical profile
ORION [34]	siRNA against PCSK-9	Inclisiran	480 patients with ASCVD or ASCVD-risk equivalents	2	Change in LDL-C from baseline to Day 180	−51%

*CAD* coronary artery disease, *MI* myocardial infarction, *CV* cardiovascular risk, *LDL-C* low-density lipoprotein cholesterol, *UAP* unstable angina pectoris, *ACS* acute coronary syndrome, *ASCVD* atherosclerotic cardiovascular disease, *PCSK-9* proprotein convertase subtilisin/kexin type-9, *siRNA* small interfering RNA, *ODYSSEY* Safety and Tolerability of Alirocumab in High Cardiovascular Risk Patients with Hypercholesterolemia Not Adequately Controlled with Their Lipid Modifying Therapy, *FOURIER* Further cardiovascular OUtcomes Research with PCSK9 Inhibition in subjects with Elevated Risk, *SPIRE* Studies of PCSK9 Inhibition and the Reduction of vascular Events, *ORION* Trial to Evaluate the Effect of ALN-PCSSC Treatment on Low-density Lipoprotein Cholesterol

The first results of two large RCTs investigating the long-term efficacy and safety of evolocumab (FOURIER trial) and alirocumab (ODYSSEY Outcomes trial) are underway and necessary [19, 20]. Recently, the development of another monoclonal PCSK-9 inhibitor, bococizumab, was stopped due to auto-antibodies formation against the compound that significantly reduced the LDL-C-lowering efficacy (The SPIRE program) [21].

#### TG-lowering therapy

##### Statins

Statins reduce the plasma concentration of TG-rich particles by inhibiting HMG-CoA reductase. Although recent evidence positions HTG as a CV risk factor, the benefits of lowering elevated TG levels are still modest.

Statins are the first-choice therapy in patients with HTG since they reduce both the CV risk and, in high doses, have a stronger effect on elevated TG levels (up to 27% reduction) [3, 22].

##### Fibrates

Fibrates are agonists of peroxisome proliferator-activated receptor-α (PPAR-α), acting via transcription factors regulating various steps in lipid and lipoprotein metabolism. Fibrates have good efficacy in lowering fasting TG as well as post-prandial TGs and TG-rich lipoprotein remnant particles, with lowering TG levels up to more than 50% [23]. However, results from 5 prospective RCTs and 5 meta-analyses failed to demonstrate superior CV outcomes with fibrates, especially when used on top of statins [3].

##### n-3 fatty acids

n-3 fatty acids (eicosapentaenoic acid (EPA) and docosahexaenoic acid (DHA)) can lower TG possibly through interaction with PPARs. Although the underlying mechanism is poorly understood n‑3 fatty acids can reduce TG levels with up to 45%. A meta-analysis of 20 studies and 63,000 patients found no overall effect of omega-3 fatty acids on composite CV events. n‑3 fatty acids appear to be safe and not interact with other therapies [24].

Currently, there are two ongoing phase 3 randomised placebo-controlled clinical trials evaluating the effect of EPA on CV outcomes in 21,000 subjects with elevated serum TG [25, 26]. If TG are not controlled by statins or fibrates n‑3 fatty acids may be added to decrease TG further, as these combinations are safe and well tolerated [3].

#### HDL-C increasing therapy

Even though lifestyle changes may increase HDL-C levels to a certain degree, many patients will also require medication should a robust HDL-C increase be considered necessary. To date, there is no convincing evidence that artificially raising HDL-C leads to an improved CV outcome. However, if HDL-C increasing therapy is considered then the following options are available.

##### Cholesteryl ester transfer protein (CETP) inhibitors

The inhibition of CETP by small molecule inhibitors represents currently the most efficient pharmacological approach to influence low HDL-C, with an effect of ≥100% increase in HDL-C and frequently a reduction of LDL-C levels as well. Despite the impressive HDL-C increase, no effect has been seen yet on CV endpoints, as all the CETP-inhibitors studies [27–29] have failed to demonstrate this thus far.

Torcetrapib was discontinued following a higher mortality in the torcetrapib arm of the ILLUMINATE trial [27], the results of the dalcetrapib trial (Dal-OUTCOMES) showed no clinical impact in acute coronary patients and the A﻿C﻿C﻿E﻿L﻿E﻿R﻿A﻿T﻿E trial of evacetrapib in acute coronary patients on statins was terminated prematurely due to lack of efficacy signals [28, 29].

Of the CETP inhibitors initially developed, only anacetrapib is still active. In mice models it has been reported that anacetrapib attenuates atherosclerosis not by increasing HDL-C but rather by decreasing LDL-C by CETP inhibition and by a CETP independent reduction of plasma PCSK-9 level [30].

The REVEAL study, a very large phase 3 RCT with anacetrapib, is still underway and its results are expected in 2017 [31]. This trial will further elucidate whether the additional beneficial effects of anacetrapib on top of a statin can be translated into clinical benefit.

##### Statins

Statins produce elevations in HDL-C levels between 5–10% [32]. It is difficult to extract the amount of effect that HDL-C increase might have in the overall observed CV risk reduction with statins.

##### Fibrates

Fibrates increase HDL-C in a similar proportion with statins, namely between 5% in long-term trials (especially if type 2 DM patients are included) and up to 15% in short-term studies [23, 33]. The FIELD study failed to demonstrate that fenofibrate could significantly lower the CV risk [23].

### Future perspectives

#### LDL-C-lowering therapy

##### PCSK-9 inhibition (non-monoclonal antibody)

A recent approach in decreasing PCSK-9 levels is the administration of small interfering RNA (siRNA) molecules directed against PCSK-9. The siRNA molecules enable the RNA-induced silencing complex, which cleaves messenger RNA (mRNA) molecules encoding PCSK-9 specifically. The cleaved mRNA is degraded and thus unavailable for protein translation, which results in decreased levels of the PCSK-9 protein. The phase 2 ORION trial showed that one subcutaneous injection of 300 mg inclisiran determined a mean LDL-C reduction of 51% after 6 months [34]. Inclisiran was well tolerated with no relevant safety concerns. These results support the start of the phase 3 program. The next step might be the development of a vaccine targeting PCSK-9. Crossey et al. provided in mice and macaques the proof-of-principle evidence that a vaccine targeting PCSK-9 peptide can effectively lower lipid levels and works synergistically with statins [35].

##### Bempedoic acid

Bempedoic acid is a first-in-class adenosine triphosphate (ATP) Citrate Lyase inhibitor. The mechanism of action involves the inhibition of cholesterol biosynthesis and the up-regulation of LDL-R, which in turn decreases plasma LDL-C levels. A phase 3 clinical trial (CLEAR Harmony) is currently conducted in patients with high CV risk and elevated LDL-C that is not adequately controlled under their current therapy. Almost 2000 subjects will be randomised for bempedoic acid or placebo and will be followed for 52 weeks [36]. In continuation of this trial, the CLEAR Outcomes trial will be conducted. This will be an event-driven study of 12,600 patients on either bempedoic acid or placebo with the primary efficacy endpoint of major adverse CV events. The results of this trial will be expected not earlier than 2022.

##### Peroxisome proliferator-activated receptor delta (PPARδ)

PPARδ is a nuclear receptor that regulates genes involved in lipid storage and transport. MBX-8025 is a selective agonist for PPARδ.

The recently presented partial results from a proof-of-concept phase II trial in patients with homozygous familial hypercholesterolaemia showed that the range of responses to MBX-8025 was broad, but that MBX-8025 could provide a clinically meaningful reduction in LDL-C for a subset of patients [37].

#### Other lipoprotein modification targets

##### Apo A-I mimetics

Apo A-I is the primary functional component of HLD-C and supports the rapid removal of cholesterol from plaque. The MILANO-PILOT study was a proof-of-concept study in which the impact on coronary plaque by MDCO-216 was measured in 120 acute coronary syndrome (ACS) patients using IVUS [38]. MDCO-216 is a complex of dimeric recombinant apolipoprotein A-I Milano and a phospholipid (POPC), and mimics pre-beta HDL. In this study, MDCO-216 did not produce a significant effect on coronary progression. Based on these results further development of the compound was halted. CER-001 is a different engineered pre-beta HDL compound and is currently being tested in a phase 2 clinical trial (CARAT) assessing the nominal change from baseline to follow-up (at 12 weeks) in the PAV in the target coronary artery of ACS patients. Results will be available in early 2017 [39]. CSL112 is a plasma-derived apolipoprotein A-I (apo A-I) and was tested in a phase II trial for safety and tolerability. CSL112 was well tolerated and did not significantly alter liver or kidney functions [40]. Assessment of the efficacy of CSL112 will be performed in an adequately powered phase 3 clinical trial.

##### Angiopoietin-like 3 (ANGPTL3)

ANGPTL3 is a protein and main regulator of lipoprotein metabolism. Its function is linked to the inhibition of lipoprotein lipase (LPL) activity. Earlier studies have identified that subjects with ANGPTL3 deficiency have reduced cholesterol and TG levels. Recently, a phase 1/2 study evaluated the safety, tolerability, pharmacokinetics and pharmacodynamics of ANGPTL3-LRx (an antisense inhibitor of ANGPTL3) in healthy volunteers with elevated TG and subjects with familial hypercholesterolaemia. There were no short-term safety concerns and ANGPTL3-LRx induced significant mean reductions in TGs (66%), LDL-C (35%) and total cholesterol (36%). Final results are expected in 2017 [41].

##### Lipoprotein(a) (Lp(a))

PCSK-9 inhibitors and nicotinic acid reduce Lp(a) by approximately 30% [16, 17, 42], however, an effect on CV events targeting Lp(a) has not been convincingly shown. A phase 2 clinical trial showed that IONIS-APO(a)Rx, an oligonucleotide targeting Lp(a), induced a lowering of Lp(a) levels of up to 71.6% [43]. A phase 1/2a first-in-man trial showed that IONIS-APO(a)-LRx, a ligand-conjugated antisense oligonucleotide designed to be highly and selectively taken up by hepatocytes, induced a lowering of Lp(a) levels of up to 92%. Both antisense oligonucleotides were short-term safe and well tolerated [43].

Plasma Lp(a) is currently not recommended for risk screening in the general population, but measurement should be considered in people with high CV risk or a strong family history of premature atherothrombotic disease [3].

Table 4 provides an overview of the most important ongoing lipoprotein modifying trials and their expected or recently published results.

**Table 4:** Ongoing trials and future perspective

Target	Clinical trial	Mechanism of action	Molecules	Population	Phase	Endpoint	Results/expected results
LDL-C	CLEAR Harmony [36]	ACL-inhibitor	Bempedoic acid	1950 high CV risk patients	3	Safety, tolerability	2018
	MBX-8025 [37]	Selective PPARδ	MBX-8025	13 patients with HoFH	2	Effect on LDL-C	Full results – early 2017
HDL-C	REVEAL [31]	CETP inhibitors	Anacetrapib	30,624 patients with a history of MI stroke or PAD	3	Major coronary events (defined as coronary death, MI or coronary revascularisation)	Early 2017
	MILANO-PILOT [38]	Apo A‑I mimetics	MDCO-216	120 ACS patients	2	Change in PAV	No significant effect
	CARAT [39]	Apo A‑I mimetics	CER-001	301 ACS patients	2	Change in PAV	Early 2017
	AEGIS [40]	Apo A‑I mimetics	CSL-112	1258 ACS patients	2b	Safety, tolerability, PK	Well tolerated and safe
Triglycerides	IONIS ANGPTL3-LRx [41]	Inhibition of LPL activity	IONIS ANGPTL3-LRx	61 healthy volunteers	1–2	Safety, tolerability, PK/PD	June 2017
L(p) a	IONIS-APO(a)-Rx [43]	Antisense oligonucleotide targeting hepatic apo(a) mRNA	IONIS-APO(a)-LRx	64 participants with high Lp(a) levels	2	%change in Lp(a)	−71.6%
	IONIS-APO(a)-LRx [43]	Ligand-conjugated antisense oligonucleotide	IONIS-APO(a)-LRx	58 healthy volunteers	1/2	%change in fasting Lp(a)	−92%

*LDL-C* low-density lipoprotein cholesterol, *ATP* adenosine triphosphate, *ACL-inhibitor* ATP-Citrate Lyase inhibitor, *PPARδ* peroxisome proliferator-activated receptor delta, *HoFH* homozygous familiar hypercholesterolemia, *CV* cardiovascular, *ACS* acute coronary syndrome, *PAV* percentage atheroma volume, *PK* pharmacokinetics, *PD* pharmacodynamics, *ApoA-I* apolipoprotein A-I, *MI* myocardial infarction, *PAD* peripheral arterial disease, *CETP* cholesteryl ester transfer protein, *LPL* lipoprotein lipase, *Lp(a)* lipoprotein (a), *mRNA* messenger RNA, *MILANO-PILOT* MDCO-216 Infusions Leading to Changes in Atherosclerosis: A Novel Therapy in Development to Improve Cardiovascular Outcomes – Proof of Concept Intravascular Ultrasound (IVUS), Lipids, and Other Surrogate Biomarkers Trial, *CARAT* CER-001 Atherosclerosis Regression ACS Trial, *AEGIS* The ApoA-I Event Reduction in Ischemic Syndromes I, *REVEAL* Randomized EValuation of the Effects of Anacetrapib though Lipid-modification, *IONIS ANGPTL3-LRx* IONIS *Angiopoietin-like 3‑linear RNAx*

## Conclusions

Lowering LDL-C by statin therapy remains, to date, the cornerstone for the medical prevention and treatment of atherosclerotic disease since it is efficient and generally safe. In high-risk patients with statin intolerance or in high-risk patients who do not obtain the desired LDL-C level with intensive statin treatment, cholesterol absorption inhibitors, especially ezetimibe, should be considered. Bile acid sequestrants, fibrates and niacin are not recommended. Upcoming PCSK-9 inhibitors, whether in the form of monoclonal antibodies or new approaches, appear as potent agents for dyslipoproteinaemia. However, their long-term efficacy and safety still needs to be proven and costs may limit their practical use. HDL-C modulation through CETP inhibition and apo A-I mimetics did not yet provide evidence for better CV outcomes; the REVEAL and CARAT trials will shed light on the future of these drug classes. New classes of molecules targeting ANGPTL3 and Lp(a) have shown promising efficacy and good short-term safety profiles in several early phase trials and these results warrant further development.
